# Presentations of perforated colonic pathology in patients with polymyalgia rheumatica: two case reports

**DOI:** 10.1186/1752-1947-4-299

**Published:** 2010-09-06

**Authors:** Punyanganie de Silva, Nagarajan Pranesh, Guy Vautier

**Affiliations:** 1 Department of Gastroenterology, James Paget University Hospital, Lowestoft Road, Great Yarmouth, NR31 6LA, UK; 2 Department of Surgery, James Paget University Hospital, Lowestoft Road, Great Yarmouth, NR31 6LA, UK; 3 Department of Gastroenterology, James Paget University Hospital, Lowestoft Road, Great Yarmouth NR31 6LA, UK

## Abstract

**Introduction:**

Polymyalgia rheumatica is an increasingly common disease in older people, which gives rise to arthralgia and is mainly treated with corticosteroids. Patients in this age group also have a higher incidence of other co-morbidities including colonic pathology. Corticosteroid usage may mask signs of sepsis or complications secondary to intra-abdominal pathology, thereby delaying diagnosis and treatment, with eventual adverse outcome. These two cases highlight the importance of awareness and prompt recognition of this condition in order to avoid significant morbidity and mortality.

**Case presentation:**

**Case 1:**

A 73-year-old Caucasian woman with a diagnosis of polymyalgia presented with symptoms of an exacerbation in her right hip joint. Despite standard therapy with corticosteroids she failed to improve and started to develop features of widespread sepsis. Specific questioning revealed that, at the very onset of her symptoms, she had experienced mild diarrheal symptoms. Investigations revealed perforated diverticular disease with a peri-femoral abscess.

**Case 2:**

A 69-year-old Caucasian woman with polymyalgia presented with left thigh pain and weakness associated with weight loss. A diagnosis of exacerbation of polymyalgia rheumatica was made and she was treated with corticosteroid therapy. Shortly afterwards she was admitted with generalized peritonitis. Laparotomy revealed a retroperitoneal abscess secondary to a perforated sigmoid colonic tumor.

**Conclusions:**

Patients with polymyalgia may have perforated colonic diverticular disease which mimics their rheumatic pathology. In such cases steroid therapy, which is the mainstay of polymyalgia therapy, can be detrimental. Primary and hospital practitioners are encouraged to be vigilant regarding non-specific gastrointestinal symptoms and consider alternative diagnoses in those patients whose symptoms do not resolve with standard therapy, as this can lead to an overall better outcome.

## Introduction

Polymyalgia rheumatica (PMR) is one of the most common chronic inflammatory conditions in elderly individuals [1]. The disease can be seen in any ethnic group, and mainly affects those over the age of 65. It is rare in people under 50 and prevalence increases with age. The incidence of the disease in patients over 50 is between 50 and 100 per 100,000 [1,2]. Symptoms can be non-specific, but usually patients present with proximal joint pain and stiffness. Previous studies have revealed that corticosteroid therapy is the only known effective treatment [3]. However, several other autoimmune, infectious, endocrine, and malignant disorders can present with similar symptoms [4]. Therefore, it is important that prior to commencing treatment in previously known or new onset cases, other potential differential diagnoses are excluded as there is a possibility that corticosteroid therapy may be detrimental. We highlight two such cases that presented in patients with known PMR.

## Case presentation

### Case 1

**Figure 1:**
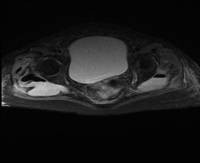
**Bilateral gluteal abscesses with gas extending around right femoral head**.

**Figure 2:**
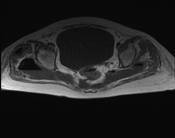
**Perforated posterior diverticulum extending into pre-sacral space**.

A 73-year-old Caucasian woman with a diagnosis of polymyalgia presented with a one month history of progressive pain and stiffness in her right hip, and myalgia of the right thigh. She had had intermittent diarrhea over the past four weeks but no bleeding or mucous appeared in her stool. Although treated as a rheumatic flare by her general practitioner, and low dose corticosteroids (prednisolone 20 mg daily) had been commenced three weeks prior to admission, she had failed to improve and therefore in-patient assessment was sought. Her past medical history consisted of hypertension and two successful normal vaginal deliveries. There was no significant family history. Apart from prednisolone, her only other medication was bendrofluazide 2.5 mg once daily. On admission to hospital, examination revealed reduced right hip and knee power of 4/5. Her diarrhea had settled by the time of admission to hospital. She was apyrexial, with a white cell count of 22.6×10^9^/dL, neutrophils 18.36, C-reactive protein (CRP) 279 mg/L and her body mass index (BMI) was 27 kg/m^2^. Hip, pelvic, chest and abdominal X-rays, ultrasound scan of the abdomen, pelvis and stool cultures were unremarkable. Sigmoidoscopy revealed mild active proctitis and diverticulosis. A diagnosis of exacerbation of PMR was made and corticosteroid dosage increased to 40 mg daily. In view of raised inflammatory markers, intravenous broad spectrum antibiotics were also commenced (Tazocin (piperacillin and tazobactam) and gentamicin). Despite this her limb weakness and hip pain became progressively worse and inflammatory markers continued to rise. Magnetic resonance imaging (MRI) of the spine and pelvis was therefore arranged on day five of admission. This revealed a posterior diverticular perforation into the pre-sacral space resulting in large bilateral gluteal abscesses. There was also gas extending around the right femoral head (Figures 1 and 2). Although an urgent percutaneous computed tomography (CT)-guided drainage with a view to proceed to laparotomy and Hartmann's procedure was arranged, she became increasingly septic and died.

### Case 2

A 69-year-old Caucasian woman with PMR presented to her general practitioner with a three-week history of left thigh pain. She also had left-sided abdominal pains for six months, which were treated with paracetamol 1 g and mebeverine 135 mg as required. Her bowels usually alternated between constipation and diarrhea with no recent change or rectal bleeding. She had reduced appetite and weight loss of 3 kg over three months. BMI was 20 kg/m^2^. Her past medical history was unremarkable apart from known polymyalgia and she had had one previous normal vaginal delivery. There was no significant family history. She was normally on 10 mg of prednisolone daily. She was on no other medication. Mild weakness of left hip flexion was noted and a white cell count 19.6×10^9^/dL, CRP 230 mg/L.

A diagnosis of exacerbation of PMR was made and her steroid dose increased to 30 mg daily. She was admitted a week later with severe abdominal pain, tachycardia and a fever of 38°C. Abdominal examination confirmed generalized peritonitis.

**Figure 3:**
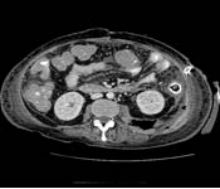
**Retroperioneal abscess secondary to perforated sigmoid tumor**.

Laparotomy revealed fecal peritonitis and a large retroperitoneal abscess due to an obstructing proximal sigmoid tumor with perforation. She underwent a Hartmann's procedure followed by a further laparotomy for a residual retroperitoneal abscess (Figure 3). Tazocin and gentamicin were administered as antibiotics. Histopathology confirmed a T4 N2 Mx moderately differentiated adenocarcinoma with incomplete resection margins. After a protracted stay in intensive care with multi-organ failure, she was discharged home and received palliative chemotherapy.

## Conclusions

Patients with polymyalgia may have perforated colonic or purulent diverticular disease which mimics their rheumatic pathology. In such cases steroid therapy which is the mainstay of polymyalgia therapy can be detrimental [5]. Although in both cases our patients' main complaint was joint/musculoskeletal pain, they also had non-specific gastrointestinal symptoms at a preceding early stage. In the presence of atypical symptoms that cannot be attributed to polymyalgia, such as diarrhea, abdominal pain or weight loss, a high degree of clinical suspicion should be maintained for an alternative primary gastrointestinal pathology.

These two cases highlight the importance of paying close attention to abdominal symptoms that cannot be attributed to polymyalgia and the need to exclude a primary intra-abdominal pathology first. Abdominal X-rays, ultrasound and sigmoidoscopy may be misleading and therefore if patients fail to improve, prompt imaging with CT/MRI is recommended in order to initiate appropriate therapy before patients become too unstable to receive treatment [6,7].

Case 1 highlights how plain film imaging may fail to detect perforations due to the absence of significant pneumoperitoneum.

## Abbreviations

BMI: body mass index; CRP: C-reactive protein; CT: computed tomography; MRI: magnetic resonance imaging; PMR: polymyalgia rheumatica.

## Consent

Written informed consent was obtained from the relatives of the patient in Case 1 for publication of this case report and accompanying images. A copy of the written consent is available for review by the journal's Editor-in-Chief. Written informed consent could not be obtained from patient 2 because the patient is now deceased and we were unable to contact a next of kin despite reasonable attempts. Every effort has been made to protect the identity of the patient and there is no reason to believe that the family would object to publication.

## Conflict of interest

The authors declare that there is no conflict of interest. No funding was sought or received for this report.

## Competing interests

The authors declare that they have no competing interests.

## Authors' contributions

PdeS was involved in the management of Case 1, and was involved in conception of the case reports, data acquisition, literature review, writing the article and critical revision. GV was involved in management of the cases, conception and critical revision. NP was involved in the management of Case 2, data acquisition and critical revision. All authors read and approved the final manuscript.
